# 5-Nitro-1-*n*-octyl-1*H*-benzimidazol-2(3*H*)-one

**DOI:** 10.1107/S1600536811003503

**Published:** 2011-02-05

**Authors:** Younes Ouzidan, Y. Kandri Rodi, Natalie Saffon, El Mokhtar Essassi, Seik Weng Ng

**Affiliations:** a Laboratoire de Chimie Organique Appliquée, Faculté des Sciences et Techniques, Université Sidi Mohamed Ben Abdallah, Fés, Morocco; b Service Commun Rayons-X FR2599, Université Paul Sabatier Bâtiment 2R1, 118 route de Narbonne, Toulouse, France; c Laboratoire de Chimie Organique Hétérocyclique, Pôle de Compétences Pharmacochimie, Université Mohammed V-Agdal, BP 1014 Avenue Ibn Batout, Rabat, Morocco; d Department of Chemistry, University of Malaya, 50603 Kuala Lumpur, Malaysia

## Abstract

The benzimidazolone part of the mol­ecule of the title compound, C_15_H_21_N_3_O_3_, is almost planar (r.m.s. deviation = 0.007 Å) with its mean plane aligned at a dihedral angle of 10.4 (3)° with respect to the mean plane of the nitro substituent. In the crystal, two mol­ecules are disposed about a center of inversion, generating an N—H⋯O hydrogen-bonded cyclic dimer with *R*_2_^2^(8) graph-set motif.

## Related literature

For the crystal structure of 1-isopropenyl-1*H*-benzimidazol-2(3*H*)-one, see: Saber *et al.* (2010 ▶). For graph-set notation, see: Etter (1990 ▶).
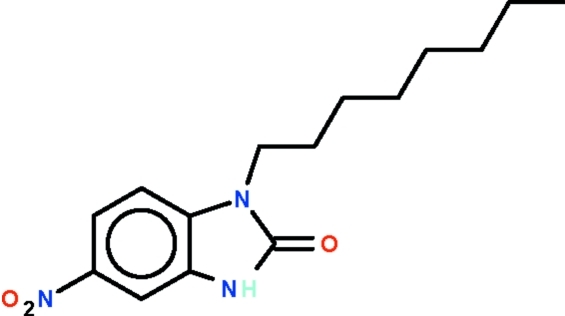


## Experimental

### 

#### Crystal data

C_15_H_21_N_3_O_3_
*M* *_r_* = 291.35
Triclinic, 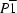

*a* = 4.9997 (3) Å
*b* = 11.4942 (6) Å
*c* = 13.8739 (7) Å
α = 74.214 (3)°
β = 79.637 (4)°
γ = 84.108 (4)°
*V* = 753.50 (7) Å^3^
*Z* = 2
Mo *K*α radiation
μ = 0.09 mm^−1^
*T* = 295 K
0.22 × 0.12 × 0.06 mm

#### Data collection

Bruker APEXII diffractometer
10215 measured reflections
3084 independent reflections
1538 reflections with *I* > 2σ(*I*)
*R*_int_ = 0.080

#### Refinement

*R*[*F* ^2^ > 2σ(*F* ^2^)] = 0.055
*wR*(*F* ^2^) = 0.144
*S* = 0.95
3084 reflections
195 parameters
H atoms treated by a mixture of independent and constrained refinement
Δρ_max_ = 0.21 e Å^−3^
Δρ_min_ = −0.17 e Å^−3^

### 

Data collection: *APEX2* (Bruker, 2005 ▶); cell refinement: *SAINT* (Bruker, 2005 ▶); data reduction: *SAINT*; program(s) used to solve structure: *SHELXS97* (Sheldrick, 2008 ▶); program(s) used to refine structure: *SHELXL97* (Sheldrick, 2008 ▶); molecular graphics: *X-SEED* (Barbour, 2001 ▶); software used to prepare material for publication: *publCIF* (Westrip, 2010 ▶).

## Supplementary Material

Crystal structure: contains datablocks global, I. DOI: 10.1107/S1600536811003503/zs2090sup1.cif

Structure factors: contains datablocks I. DOI: 10.1107/S1600536811003503/zs2090Isup2.hkl

Additional supplementary materials:  crystallographic information; 3D view; checkCIF report

## Figures and Tables

**Table 1:** Hydrogen-bond geometry (Å, °)

*D*—H⋯*A*	*D*—H	H⋯*A*	*D*⋯*A*	*D*—H⋯*A*
N2—H1⋯O1^i^	0.93 (3)	1.84 (3)	2.755 (3)	169 (3)

Symmetry code: (i) 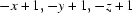.

**Fig. 1.**
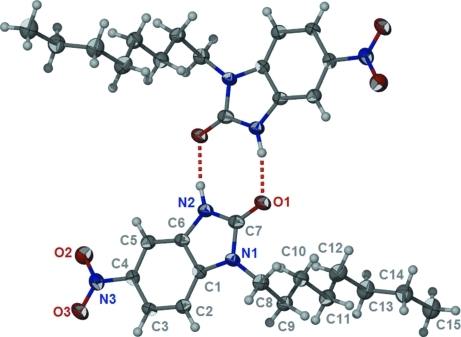
Thermal ellipsoid plot (Barbour, 2001) of two molecules of C15H21N3O3 disposed about a center of inversion: drawn at the 50% probability level.

## supplementary crystallographic information

### Comment

Tetraalkylammonium halides are used as phase-transfer catalyst in the synthesis of alkyl-substituted benzimidazolones. A previous study reported the 1-isopropenyl derivative; the amino –NH unit forms a hydrogen bond to the inversion-related molecule to generate a hydrogen-bonded dimer (Saber *et al.*, 2010). The present compound (Scheme I) features a long *n*-octyl chain that adopts an extended zigzag conformation (Fig. 1). The benzimidazolone part of the C_15_H_21_N_3_O_3_ molecule is planar (r.m.s. deviation 0.007 Å) and its mean plane is aligned at 10.4 (3) ° with respect to the mean plane of the nitro substituent. Two molecules are disposed about a center of inversion to generate a hydrogen-bonded cyclic dimer, whose hydrogen-bonding motif is described by the *R*_2_^2^(8) graph set (Etter, 1990).

### Experimental

To 5-nitro-1*H*-benzoimidazol-2(3*H*)-one (0.2 g, 1.1 mmol), potassium carbonate (0.30 g, 2.2 mmol) and tetra-*n*-butylammonium bromide (0.07 g, 0.2 mmol) in DMF (15 ml) was added 1-bromo-*n*-octane (0.38 ml, 2.2 mmol). Stirring was continued at room temperature for 6 h. The salt was removed by filtration and the filtrate concentrated under reduced pressure. The residue was separated by chromatography on a column of silica gel with ethyl acetate/hexane (1/2) as eluent. The compound was recrystallized from diethyl ether to give colorless crystals.

### Refinement

Carbon-bound H-atoms were placed in calculated positions (C—H 0.93–0.97 Å) and were included in the refinement in the riding model approximation, with *U*_iso_(H) set to 1.2–1.5*U*_eq_(C).

### Crystal data

**Table:**

C_15_H_21_N_3_O_3_	*Z* = 2
*M**_r_* = 291.35	*F*(000) = 312
Triclinic, *P*1	*D*_x_ = 1.284 Mg m^−^^3^
Hall symbol: -P 1	Mo *K*α radiation, λ = 0.71073 Å
*a* = 4.9997 (3) Å	Cell parameters from 926 reflections
*b* = 11.4942 (6) Å	θ = 2.1–26.5°
*c* = 13.8739 (7) Å	µ = 0.09 mm^−^^1^
α = 74.214 (3)°	*T* = 295 K
β = 79.637 (4)°	Block, colorless
γ = 84.108 (4)°	0.22 × 0.12 × 0.06 mm
*V* = 753.50 (7) Å^3^	

### Data collection

**Table:**

Bruker APEXII diffractometer	1538 reflections with *I* > 2σ(*I*)
Radiation source: fine-focus sealed tube	*R*_int_ = 0.080
graphite	θ_max_ = 26.5°, θ_min_ = 2.1°
φ and ω scans	*h* = −5→6
10215 measured reflections	*k* = −14→14
3084 independent reflections	*l* = −17→17

### Refinement

**Table:**

Refinement on *F*^2^	Secondary atom site location: difference Fourier map
Least-squares matrix: full	Hydrogen site location: inferred from neighbouring sites
*R*[*F*^2^ > 2σ(*F*^2^)] = 0.055	H atoms treated by a mixture of independent and constrained refinement
*wR*(*F*^2^) = 0.144	*w* = 1/[σ^2^(*F*_o_^2^) + (0.0609*P*)^2^] where *P* = (*F*_o_^2^ + 2*F*_c_^2^)/3
*S* = 0.95	(Δ/σ)_max_ = 0.001
3084 reflections	Δρ_max_ = 0.21 e Å^−^^3^
195 parameters	Δρ_min_ = −0.17 e Å^−^^3^
0 restraints	Extinction correction: *SHELXL97* (Sheldrick, 2008), Fc^*^=kFc[1+0.001xFc^2^λ^3^/sin(2θ)]^-1/4^
Primary atom site location: structure-invariant direct methods	Extinction coefficient: 0.016 (4)

### Fractional atomic coordinates and isotropic or equivalent isotropic displacement parameters (Å^2^)

**Table:**

	*x*	*y*	*z*	*U*_iso_*/*U*_eq_	
O1	0.6268 (4)	0.47630 (17)	0.37325 (14)	0.0426 (5)	
O2	−0.5372 (4)	0.15689 (19)	0.72705 (15)	0.0534 (6)	
O3	−0.5678 (4)	0.02018 (19)	0.64945 (15)	0.0534 (6)	
N1	0.3626 (4)	0.32787 (19)	0.35991 (16)	0.0325 (6)	
N2	0.2757 (5)	0.3907 (2)	0.50001 (18)	0.0351 (6)	
N3	−0.4666 (5)	0.1112 (2)	0.65519 (18)	0.0407 (6)	
C1	0.1487 (5)	0.2628 (2)	0.42194 (19)	0.0299 (6)	
C2	0.0021 (5)	0.1746 (2)	0.4085 (2)	0.0341 (7)	
H2	0.0387	0.1491	0.3491	0.041*	
C3	−0.2003 (5)	0.1257 (2)	0.48628 (19)	0.0336 (7)	
H3	−0.3034	0.0660	0.4800	0.040*	
C4	−0.2502 (5)	0.1658 (2)	0.57430 (19)	0.0324 (6)	
C5	−0.1062 (5)	0.2549 (2)	0.58994 (19)	0.0339 (7)	
H5	−0.1433	0.2802	0.6494	0.041*	
C6	0.0937 (5)	0.3025 (2)	0.51182 (19)	0.0316 (7)	
C7	0.4414 (6)	0.4067 (3)	0.4080 (2)	0.0364 (7)	
C8	0.4867 (6)	0.3212 (3)	0.25805 (19)	0.0399 (7)	
H8A	0.5128	0.2370	0.2563	0.048*	
H8B	0.6644	0.3549	0.2417	0.048*	
C9	0.3124 (6)	0.3898 (2)	0.17815 (19)	0.0381 (7)	
H9A	0.4130	0.3908	0.1113	0.046*	
H9B	0.1488	0.3469	0.1872	0.046*	
C10	0.2313 (6)	0.5199 (2)	0.1835 (2)	0.0407 (7)	
H10A	0.1183	0.5181	0.2484	0.049*	
H10B	0.3947	0.5602	0.1806	0.049*	
C11	0.0788 (6)	0.5937 (3)	0.0999 (2)	0.0429 (8)	
H11A	−0.0721	0.5490	0.0964	0.051*	
H11B	0.1997	0.6062	0.0354	0.051*	
C12	−0.0298 (6)	0.7161 (3)	0.1179 (2)	0.0491 (8)	
H12A	0.1215	0.7581	0.1248	0.059*	
H12B	−0.1543	0.7024	0.1816	0.059*	
C13	−0.1770 (6)	0.7985 (3)	0.0349 (2)	0.0507 (9)	
H13A	−0.3121	0.7528	0.0213	0.061*	
H13B	−0.2729	0.8647	0.0601	0.061*	
C14	0.0045 (6)	0.8505 (3)	−0.0629 (2)	0.0509 (8)	
H14A	0.0916	0.7847	−0.0908	0.061*	
H14B	0.1463	0.8924	−0.0492	0.061*	
C15	−0.1477 (7)	0.9384 (3)	−0.1419 (2)	0.0608 (10)	
H15A	−0.0220	0.9683	−0.2027	0.091*	
H15B	−0.2301	1.0050	−0.1156	0.091*	
H15C	−0.2864	0.8972	−0.1568	0.091*	
H1	0.296 (6)	0.428 (3)	0.549 (2)	0.059 (10)*	

### Atomic displacement parameters (Å^2^)

**Table:**

	*U*^11^	*U*^22^	*U*^33^	*U*^12^	*U*^13^	*U*^23^
O1	0.0398 (12)	0.0451 (12)	0.0468 (12)	−0.0145 (11)	−0.0018 (10)	−0.0170 (10)
O2	0.0546 (14)	0.0572 (14)	0.0491 (13)	−0.0085 (12)	0.0087 (11)	−0.0240 (11)
O3	0.0546 (14)	0.0502 (14)	0.0571 (14)	−0.0244 (12)	0.0059 (11)	−0.0184 (11)
N1	0.0301 (13)	0.0333 (13)	0.0366 (13)	−0.0044 (11)	−0.0056 (11)	−0.0123 (10)
N2	0.0323 (13)	0.0398 (14)	0.0369 (14)	−0.0067 (11)	−0.0038 (11)	−0.0153 (11)
N3	0.0350 (14)	0.0398 (15)	0.0446 (15)	−0.0036 (12)	−0.0038 (12)	−0.0074 (12)
C1	0.0253 (15)	0.0291 (15)	0.0350 (15)	−0.0002 (13)	−0.0035 (12)	−0.0089 (12)
C2	0.0343 (16)	0.0342 (16)	0.0353 (15)	0.0021 (13)	−0.0053 (13)	−0.0129 (12)
C3	0.0337 (16)	0.0302 (15)	0.0402 (16)	−0.0038 (13)	−0.0076 (13)	−0.0127 (13)
C4	0.0276 (15)	0.0311 (15)	0.0357 (15)	−0.0002 (13)	−0.0033 (12)	−0.0058 (12)
C5	0.0327 (16)	0.0371 (16)	0.0327 (15)	0.0001 (14)	−0.0044 (13)	−0.0119 (13)
C6	0.0306 (15)	0.0303 (15)	0.0371 (16)	0.0002 (13)	−0.0102 (13)	−0.0115 (12)
C7	0.0348 (17)	0.0355 (16)	0.0426 (17)	−0.0001 (14)	−0.0136 (14)	−0.0123 (14)
C8	0.0335 (16)	0.0457 (18)	0.0409 (17)	−0.0014 (14)	−0.0004 (13)	−0.0159 (14)
C9	0.0344 (16)	0.0467 (18)	0.0353 (16)	−0.0033 (14)	0.0021 (13)	−0.0186 (13)
C10	0.0399 (17)	0.0428 (17)	0.0394 (16)	−0.0041 (14)	−0.0029 (14)	−0.0121 (13)
C11	0.0412 (17)	0.0477 (18)	0.0393 (16)	−0.0004 (15)	−0.0021 (14)	−0.0142 (14)
C12	0.055 (2)	0.0479 (19)	0.0447 (18)	0.0084 (16)	−0.0101 (15)	−0.0150 (15)
C13	0.051 (2)	0.051 (2)	0.0470 (18)	0.0069 (16)	−0.0025 (16)	−0.0134 (15)
C14	0.0452 (19)	0.054 (2)	0.0514 (19)	−0.0034 (16)	−0.0071 (16)	−0.0098 (16)
C15	0.063 (2)	0.061 (2)	0.053 (2)	−0.0012 (19)	−0.0071 (18)	−0.0094 (17)

### Geometric parameters (Å, °)

**Table:**

O1—C7	1.230 (3)	C9—C10	1.526 (4)
O2—N3	1.231 (3)	C9—H9A	0.9700
O3—N3	1.235 (3)	C9—H9B	0.9700
N1—C7	1.385 (3)	C10—C11	1.515 (4)
N1—C1	1.388 (3)	C10—H10A	0.9700
N1—C8	1.457 (3)	C10—H10B	0.9700
N2—C7	1.368 (3)	C11—C12	1.524 (4)
N2—C6	1.389 (3)	C11—H11A	0.9700
N2—H1	0.93 (3)	C11—H11B	0.9700
N3—C4	1.464 (3)	C12—C13	1.527 (4)
C1—C2	1.380 (4)	C12—H12A	0.9700
C1—C6	1.414 (3)	C12—H12B	0.9700
C2—C3	1.378 (4)	C13—C14	1.500 (4)
C2—H2	0.9300	C13—H13A	0.9700
C3—C4	1.392 (4)	C13—H13B	0.9700
C3—H3	0.9300	C14—C15	1.529 (4)
C4—C5	1.394 (4)	C14—H14A	0.9700
C5—C6	1.369 (4)	C14—H14B	0.9700
C5—H5	0.9300	C15—H15A	0.9600
C8—C9	1.530 (3)	C15—H15B	0.9600
C8—H8A	0.9700	C15—H15C	0.9600
C8—H8B	0.9700		
			
C7—N1—C1	109.6 (2)	C10—C9—H9B	108.9
C7—N1—C8	123.1 (2)	C8—C9—H9B	108.9
C1—N1—C8	127.3 (2)	H9A—C9—H9B	107.8
C7—N2—C6	110.3 (2)	C11—C10—C9	114.5 (2)
C7—N2—H1	123.8 (19)	C11—C10—H10A	108.6
C6—N2—H1	125.6 (19)	C9—C10—H10A	108.6
O2—N3—O3	122.7 (2)	C11—C10—H10B	108.6
O2—N3—C4	118.5 (3)	C9—C10—H10B	108.6
O3—N3—C4	118.8 (2)	H10A—C10—H10B	107.6
C2—C1—N1	131.6 (2)	C10—C11—C12	111.7 (2)
C2—C1—C6	121.5 (2)	C10—C11—H11A	109.3
N1—C1—C6	106.8 (2)	C12—C11—H11A	109.3
C3—C2—C1	117.6 (3)	C10—C11—H11B	109.3
C3—C2—H2	121.2	C12—C11—H11B	109.3
C1—C2—H2	121.2	H11A—C11—H11B	107.9
C2—C3—C4	119.8 (3)	C11—C12—C13	115.1 (2)
C2—C3—H3	120.1	C11—C12—H12A	108.5
C4—C3—H3	120.1	C13—C12—H12A	108.5
C3—C4—C5	123.8 (3)	C11—C12—H12B	108.5
C3—C4—N3	118.1 (3)	C13—C12—H12B	108.5
C5—C4—N3	118.0 (2)	H12A—C12—H12B	107.5
C6—C5—C4	115.4 (2)	C14—C13—C12	114.8 (2)
C6—C5—H5	122.3	C14—C13—H13A	108.6
C4—C5—H5	122.3	C12—C13—H13A	108.6
C5—C6—N2	131.8 (3)	C14—C13—H13B	108.6
C5—C6—C1	121.7 (3)	C12—C13—H13B	108.6
N2—C6—C1	106.5 (2)	H13A—C13—H13B	107.6
O1—C7—N2	127.7 (3)	C13—C14—C15	113.2 (2)
O1—C7—N1	125.5 (3)	C13—C14—H14A	108.9
N2—C7—N1	106.8 (3)	C15—C14—H14A	108.9
N1—C8—C9	112.1 (2)	C13—C14—H14B	108.9
N1—C8—H8A	109.2	C15—C14—H14B	108.9
C9—C8—H8A	109.2	H14A—C14—H14B	107.8
N1—C8—H8B	109.2	C14—C15—H15A	109.5
C9—C8—H8B	109.2	C14—C15—H15B	109.5
H8A—C8—H8B	107.9	H15A—C15—H15B	109.5
C10—C9—C8	113.2 (2)	C14—C15—H15C	109.5
C10—C9—H9A	108.9	H15A—C15—H15C	109.5
C8—C9—H9A	108.9	H15B—C15—H15C	109.5
			
C7—N1—C1—C2	−179.8 (3)	C2—C1—C6—C5	0.4 (4)
C8—N1—C1—C2	2.2 (4)	N1—C1—C6—C5	−179.3 (2)
C7—N1—C1—C6	−0.2 (3)	C2—C1—C6—N2	−179.9 (2)
C8—N1—C1—C6	−178.2 (2)	N1—C1—C6—N2	0.5 (3)
N1—C1—C2—C3	179.3 (2)	C6—N2—C7—O1	−179.2 (3)
C6—C1—C2—C3	−0.3 (3)	C6—N2—C7—N1	0.4 (3)
C1—C2—C3—C4	0.0 (4)	C1—N1—C7—O1	179.5 (2)
C2—C3—C4—C5	0.1 (4)	C8—N1—C7—O1	−2.4 (4)
C2—C3—C4—N3	−179.8 (2)	C1—N1—C7—N2	−0.1 (3)
O2—N3—C4—C3	−170.3 (2)	C8—N1—C7—N2	177.9 (2)
O3—N3—C4—C3	10.9 (3)	C7—N1—C8—C9	−100.3 (3)
O2—N3—C4—C5	9.7 (3)	C1—N1—C8—C9	77.4 (3)
O3—N3—C4—C5	−169.1 (2)	N1—C8—C9—C10	52.0 (3)
C3—C4—C5—C6	0.0 (4)	C8—C9—C10—C11	175.1 (2)
N3—C4—C5—C6	179.9 (2)	C9—C10—C11—C12	172.1 (3)
C4—C5—C6—N2	−179.9 (2)	C10—C11—C12—C13	177.7 (3)
C4—C5—C6—C1	−0.2 (3)	C11—C12—C13—C14	−71.2 (4)
C7—N2—C6—C5	179.2 (3)	C12—C13—C14—C15	−176.5 (3)
C7—N2—C6—C1	−0.6 (3)		

### Hydrogen-bond geometry (Å, °)

**Table:**

*D*—H···*A*	*D*—H	H···*A*	*D*···*A*	*D*—H···*A*
N2—H1···O1^i^	0.93 (3)	1.84 (3)	2.755 (3)	169 (3)

Symmetry codes: (i) −*x*+1, −*y*+1, −*z*+1.
